# Effect of Poly(L-lysine) and Heparin Coatings on the Surface of Polyester-Based Particles on Prednisolone Release and Biocompatibility

**DOI:** 10.3390/pharmaceutics13060801

**Published:** 2021-05-27

**Authors:** Abdelrahman Mohamed, Viktor Korzhikov-Vlakh, Nan Zhang, André Said, Iuliia Pilipenko, Monika Schäfer-Korting, Christian Zoschke, Tatiana Tennikova

**Affiliations:** 1 Institute of Chemistry, St. Petersburg State University, Universitetskii Pr. 26, Peterhoff, 198504 St. Petersburg, Russia; st059429@student.spbu.ru (A.M.); yulia-sobinina@ya.ru (I.P.); tennikova@mail.ru (T.T.); 2 Chemistry Department, Faculty of Science, Beni-Suef University, Salah Salem Street, Beni Suef 62511, Egypt; 3 Institute of Pharmacy (Pharmacology & Toxicology), Freie Universität Berlin, Königin-Luise-Str. 2+4, 14195 Berlin, Germany; nan.zhang@fu-berlin.de (N.Z.); andre.said@fu-berlin.de (A.S.); monika.schaefer-korting@fu-berlin.de (M.S.-K.); christian.zoschke@fu-berlin.de (C.Z.); 4 Department of Veterinary Drugs, Federal Office of Consumer Protection and Food Safety, Mauerstr. 39-42, 10117 Berlin, Germany

**Keywords:** poly(ε-caprolactone), poly(L-lactide), polymeric nanoparticle, alternative methods to animal testing, biocompatibility, eye diseases, irritation, ocular drug delivery

## Abstract

A plethora of micro- and nanoparticle types are currently investigated for advanced ocular treatment due to improved drug retention times, higher bioavailability and better biocompatibility. Yet, comparative studies of both physicochemical and toxicological performance of these novel drug delivery systems are still rare. Herein, poly(L-lactic acid)- and poly(ε-caprolactone)-based micro- and nanoparticles were loaded with prednisolone as a model drug. The physicochemical properties of the particles were varied with respect to their hydrophilicity and size as well as their charge and the effect on prednisolone release was evaluated. The particle biocompatibility was assessed by a two-tier testing strategy, combining the EpiOcular^TM^ eye irritation test and bovine corneal opacity and permeability assay. The biodegradable polyelectrolyte corona on the particles’ surface determined the surface charge and the release rate, enabling prednisolone release for at least 30 days. Thereby, the prednisolone release process was mainly governed by molecular diffusion. Finally, the developed particle formulations were found to be nontoxic in the tested range of concentrations.

## 1. Introduction

Nanotechnology is currently attracting attention for improving the treatment of ocular anterior and posterior segments disorders [1,2,3]. Thereby topical, intravitreal, subconjunctival, and systemic administrations appear feasible [4]. Topical treatment is the most convenient route and is most frequently used for conventional formulations in ophthalmology [5,6]. However, physiological barriers in the eye and fast clearance of applied drugs limit the intraocular bioavailability and ask for novel drug delivery systems [7].

Different polymeric nanoparticles have been intensively studied for ocular drug delivery, such as polymeric nanospheres, nanogels, micelles or liposomes, owing to their tunable physiochemical properties and loading capacities [5]. Among such a broad spectrum of particulate formulations, polyester-based particles, such as poly(L-lactic acid) (PLA) and poly(ε-caprolactone) (PCL), are advantageous due to high drug loading and their ability to increase drug retention time in the precorneal area, with encapsulated hydrophilic and lipophilic drugs, such as sparfloxacin and brimonidine [8,9,10,11]. Polyester-based biodegradable particles hold great potential in helping to mask drug irritation to the eye, increase drug loading, prolong the precorneal retention time of the drug, improve the penetration of drugs across anatomical and physiological barriers in the eye to reach the inner anterior segment [8,9,10,11,12,13]. However, the release profile of drugs from polyesters usually possess an initial burst release stage. The intensity of this burst release depends on particle hydrophobicity and crystallinity [14]. Surface composition and particle size are known to be critical for control over their storage stability [15], early elimination from the blood by the phagocytosis process, and for particle interactions with cells [16,17]. Moreover, the surface composition could be an important factor in the determination of drug release kinetics [17,18]. Despite quite broad research on polyester-based nanoparticles, the systematic studies aimed to reveal the effect of different particle parameters on their drug release properties [14] and biointeractions [19] are met quite rarely.

The classical studies of Yuri Lvov, Orlin Velev and Vesselin Paunov showed the great impact of layer-by-layer modification of particulate formulations on their biomedical properties [20,21]. For example, these approaches allow the construction of layers of nanocarriers for the treatment of multi-drug resistant ovarian cancer [22], construct GFP-reporter yeast for micro-screening systems [23], as well as fabricate yeastosomes via templating of microbubbles with cells coated with cationic polyelectrolyte and the layer-by-layer technique [24].

In our previous study, the impact of size and surface charge of polyester-based particles on their biointeractions and intracellular uptake into different cell types was studied [19]. Luminescent PLA and PCL micro- and nanoparticles with different surface charges were prepared to track the intracellular penetration of particles. For this, carboxylic groups on the surface of particles were put into reaction with ε-aminogroups of poly(L-lysine) (PLys), resulting in positively charged particles. The species obtained were then covered with heparin (Hep) to prepare the particles bearing a negative surface charge. These particulate formulations with variable hydrophobicity, sizes and charges are interesting candidates for a comparative study of drug release kinetics.

Corticosteroids are extensively used medications in treating numerous inflammatory diseases affecting the eye’s anterior segment, including conjunctivitis [25] and uveitis [26]. Previous reports revealed that corticosteroids’ incorporation into polyester micro- and nanoparticles could effectively sustain the drug release and significantly advance its transcorneal permeation compared to marketed forms [12,13]. Thus, corticosteroids, such as prednisolone, could be considered as actual model drugs for the development of formulations capable of prolonged drug release [27].

Although PLA and PCL particulate formulations are quite well studied, there is still no clear understanding of their interaction within biological systems. Moreover, reliable in vitro methods, which can be used to evaluate their in vivo toxicity, are in demand. The generally accepted animal test for evaluating the eye toxicity of a formulation, namely, Draize rabbit eye assay [28], usually causes pain and distress to the tested animals. Besides the main ethical issues, animal testing has additional drawbacks, including manual labor content, time-consuming protocols, and high costs. Therefore, alternatives to animal testing are required to overcome these drawbacks [29]. A 2-tier non-animal testing strategy composed of the EpiOcular^TM^ Eye Irritation Test (EpiOcular^TM^-EIT; OECD TG492) and the Bovine Corneal Opacity and Permeability (BCOP; OECD TG437) assay has been established [30] with improved relevance. However, only nanomaterials, such as metal, amorphous, and carbon nanotubes, have been tested.

Herein, we loaded prednisolone to PLA and PCL micro- and nanoparticles. PLys and Hep were acquired respectively for modification of the particle surface to form the hydrophilic layers with a different charge and thickness [31]. We have shown that release of the drug could be severely affected by the coating of the particles with polyelectrolyte layers. We have also adopted and extended a human cell-based two-tier testing strategy for the evaluation of polymeric micro- and nanocarriers. To the best of our knowledge, this is the first systematic study that compares the impact of size and hydrophilicity/hydrophobicity of the particles, and the surface charge of a series of polyester-based particles on drug-release kinetics and biological properties. Overall, our findings will be useful not solely for ocular delivery, but also for a broad list of therapeutic applications of polyester-based particulate formulations.

## 2. Materials and Methods

### 2.1. Materials

PLA (M_w_ = 6400) and PCL (M_w_ = 17,500) were synthesized by ring-opening polymerization of corresponding monomers as previously described [14,19]. All reagents for particles preparation and modification: sodium hydroxide, 1-ethyl-3-(3-dimethylaminopropyl) carbodiimide (EDC), N-hydroxysuccinimide (NHS), PLys (M_w_ = 15,000–30,000), Hep from porcine intestinal mucosa and prednisolone, as well as all buffer salts, were purchased from Fluka (Buchs, Switzerland) and Sigma (Darmstadt, Germany) and used without additional purification.

### 2.2. Biological Material

The use of human cells was approved by the ethics committee (ethical approvals EA1/081/13, EA1/345/14). Normal human keratinocytes (NHK) were isolated from juvenile foreskin after the parents signed the informed consent. NHK was expanded in a low calcium keratinocyte growth medium (KGM BulletKit, Lonza, Cologne, Germany). Red blood cells (RBCs) were obtained from buffy-coat donations from anonymous healthy volunteers (German Red Cross, DRK-Blutspendedienst Ost, Berlin, Germany).

Bovine eyes for assessment of irritation potential were obtained from freshly slaughtered cattle (Teterower Fleisch, Teterow, Germany) after approval of the veterinary inspection office (DE 11 006 0007 21). EpiOcular™ kits (OCL-200) consisting of 24 tissues were purchased and delivered by MatTek Corporation (Bratislava, Slovakia).

### 2.3. Methods

#### 2.3.1. Particle Preparation

Single emulsion. PLA and PCL microparticles (MPs), loaded with a prednisolone, were prepared by a modified single emulsion evaporation according to the previously published protocol [14]. Shortly, 5 mg of drug and 100 mg of the corresponding polymer were dissolved in 4 mL of chloroform and dispersed into 80 mL of an ice-cold water phase via the simultaneous action of an ultrasound homogenizer and a magnetic stirrer at 800 rpm. The water phase contained 0.5% [*w*/*v*] sodium dodecyl sulfate (SDS) and 1% [*w*/*v*] polyvinyl alcohol (PVA). Next, the chloroform was evaporated with a rotary evaporator at 100 mbar for 2 h and then at 10 mbar for 1 h to form the particle suspension. The particles were separated with a centrifuge at 10,000× *g*, washed three times with distilled water, and freeze-dried.

Nanoprecipitation. PLA and PCL nanoparticles, loaded with prednisolone, were obtained by nanoprecipitation according to a previously published protocol [31]. Shortly, a solution of 100 mg of PLA or PCL and 5 mg of drugs in 20 mL of THF was slowly added to 100 mL of water. The mixture was stirred for one day in an opened flask to evaporate the organic solvent. The obtained suspension was freeze-dried.

**Figure 1:**
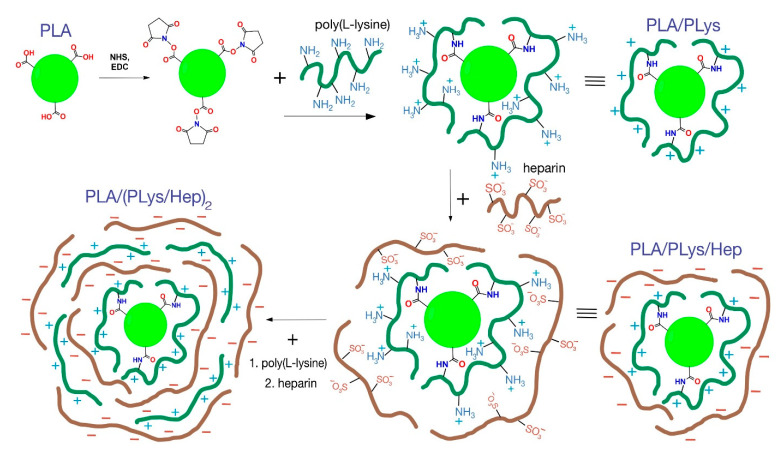
Scheme of particle modification by PLys and Hep. Note: The scales of particles and layers are not correct. The scheme shows the principal processes of particle modification.

Modification of micro-and nanoparticles with PLys and Hep. Similar to the previously published protocol, the micro- and nanoparticles were modified with PLys and Hep [19]. The scheme of particle step-by-step modification is presented in Figure 1.

Formation of MPs/NPs-PLys. The 50 mg of dry particles were resuspended in 0.5 mL of 0.01 M NaOH and kept at room temperature for 20 min. Then, the suspension was centrifuged for 5 min at 12,000× *g* and washed with distilled water. The washed particles were resuspended in 0.5 mL of EDC 1 mg/mL solution in 0.1 M MES-buffer, pH 5.5. The suspension was cooled down to 5 ℃ and mixed with 0.5 mL of NHS 1 mg/mL solution at the same buffer. The reaction mixture obtained was kept at 5 °C for 1 h, then centrifuged for 5 min at 12,000× *g* and resuspended in 1 mL of PLys 0.5 mg/mL solution in 0.01 M borate buffer, pH 8.4. The suspension was stirred at 750 rpm and at room temperature for 1 h. Next, the particles were separated via centrifugation as described above, washed twice by distilled water, and freeze-dried.

Formation of MPs/NPs-PLys-Hep. The surface of the species was further modified with Hep to inverse their charge from a positive to a negative value as follows: 20 mg of particles covered by PLys were resuspended in 0.5 mL of bidistilled water and mixed with 0.5 mL of 1 mg/mL Hep solution. The mixture was stirred at 750 rpm and room temperature during 30 min, then washed by centrifugation as described above, and freeze-dried.

Formation of MPs/NPs-(PLys-Hep)_2_. To form the second interpolyelectrolyte layer on the surface of particles covered with PLys-Hep layer, 20 mg of those were immersed sequentially into PLys and Hep 1 mg/mL solutions in bidistilled water. The intermediate MPs/NPs-PLys-Hep-PLys and final MPs/NPs-PLys-Hep-PLys-Hep = MPs/NPs-(PLys-Hep)_2_ products were separated and washed by centrifugation as described above.

#### 2.3.2. Evaluation of Particles Physicochemical Properties, Drug Entrapment Efficiency and Release Kinetics

The particles’ mean hydrodynamic diameter and size distribution were determined by dynamic light scattering (DLS), while mean ζ-potential was detected by electrophoretic light scattering (ELS). The Zetasizer Nano ZS (Malvern, UK) was applied for these measurements. The morphology of dry particles was studied by scanning electron microscopy (SEM) and scanning-transmission electron microscopy (STEM) with application of scanning electron microscopes Hitachi S-3400N and Hitachi SU8010 (Hitachi, Japan), correspondingly.

Entrapment efficiency and model drug release evaluation. Dry particles were weighted (*W_particles_*) and dissolved in DMSO to evaluate the entrapped drug quantity (*Q_entrap_*) via spectrophotometric detection. The drug loading (*DL*, μg of drug per mg of particles) and encapsulation efficiency (*EE*, %) were calculated as follows:

*DL*(μg/mg) = *Q_entrap_*/*W_particles_* (1)

*EE*(%) = *DL*/*DL*_0_ × 100 (2)

The *DL*_0_ is the theoretical quantity of entrapped drug:

*DL*_0_(μg/mg) = *C*_0_ × *V*_0_/*W_particles_* (3)

where *C*_0_ is the initial concentration of the drug solution and *V*_0_ is the volume of this solution taken for entrapment.

The release was performed in 0.01 M phosphate-buffered saline (PBS), pH 7.4, containing 0.1% of SDS. The cumulative dye release (%) was evaluated as the total quantity of drug detected in the supernatant at time t referred to the amount of initially entrapped drug.

DDSolver, a free software program, was applied for approximating the kinetic patterns of the initial and modified polyester particles with the application of zero-order, first-order, Higuchi, Hixson-Crowell, Baker-Lonsdale, Hopfenberg, Korsmeyer–Peppas, Peppas-Sahlin and Weibull models to elucidate the release mechanism of the encapsulated drug [32].

To evaluate and compare the effect of polyester modification with polyelectrolyte layers on the surface hydrophobicity, the contact angles of corresponding films (PLA, PCL, PLA-PLys, PCL-PLys, PLA-PLys-Hep, PCL-PLys-Hep, PLA-(PLys-Hep)_2_, PCL-(PLys-Hep)_2_) were measured. The films were prepared and characterized as described before [33]. Briefly, a 1.5 µL water drop was placed on the film surface, and the average static contact angle was determined using the drop analysis plugin (Biomedical Imaging Group, Lausanne, Switzerland) for ImageJ software during the first 30 s following the drop deposition. For each sample, ten measurements were done on different areas of the film. Three samples were characterized for each film composition.

### 2.4. Cytotoxicity

The cytotoxicity of the particles towards epithelial cells was determined by a 3-(4,5-dimethylthiazol-2-yl)-2,5-diphenyltetrazolium bromide (MTT) reduction assay. In brief, NHK was seeded into 96-well plates, and (co)polymer nanoparticles were added at 0.05% and 0.005% [*w*/*v*] in KGM for 48 h; 100 μL MTT solution (5 mg/mL in PBS) was added after the incubation period for 4 h. The absorbance was measured at 540 nm (FLUOstar Optima, BMG Labtech, Offenburg, Germany). All experiments were performed in triplicate. For toxicity assessment, KGM was used as a reference for untreated cells; the addition of 0.005% [*w*/*v*] sodium dodecyl sulfate served as a positive control, 10% [*v*/*v*] distilled water as a solvent control. The data are presented as relative viability. The mean value of solvent control (corrected for blank value) was set to 100%. Cell viability below 75% indicates cytotoxic effects [34].

*Viability* (%) = *corrected testing nanocarrier OD*_540_/*corrected solvent control OD*_540_ × 100 (4)

### 2.5. Irritation Potential

A red blood cell (RBC) test was performed according to INVITTOX N°37 protocol [35]. RBC diluted with 10 mM glucose (Sigma-Aldrich) to 4 × 109 cells/mL was added to nanoparticles in PBS. Deionized water served as a positive control, PBS as a negative control, and SDS solutions ranging in concentrations from 0 to 80 ppm as a hemolysis reference. The absorbance of the released oxyhemoglobin at 560 nm (WPA Biowave, Biochrom, Berlin, Germany) was measured, and the relation between the effective concentration of 50% hemolysis and protein denaturation was calculated to evaluate the acute eye irritancy potential.

A bovine corneal opacity and permeability (BCOP) test was performed according to OECD guideline no. 437 [36]. Briefly, the cornea of bovine eyes was isolated and washed in Hanks’ Balanced Salt Solution (HBSS with Ca^2+^ and Mg^2+^) mounted in the cornea holders with Minimum Essential Medium (MEM, without Phenol Red; both from Gibco by Life Technologies^TM^, Carlsbad, CA, USA) and measured for initial opacity after 1 h (Opacitometer Kit, BASF-OP 3.0, Ludwigshafen, Germany). Nanoparticles 0.05% [*w*/*v*] were added for a period of 10 min, and the final opacity was measured after 4 h. Permeability was determined by adding sodium Fluorescein solution (4 mg/mL, Sigma-Aldrich). Spectrophotometric measurements (WPA Biowave, Biochrom, Cambridge, UK) evaluated at 490 nm were recorded as optical density (*OD*_490_). De-ionized water was used as a negative control, and 100% [*v*/*v*] ethanol (VWR, Darmstadt, Germany) as a positive control. The final in vitro irritancy score (*IVIS*) was calculated with the equation

*IVIS* = *mean opacity value* + (15 × *mean permeability OD*_490_*value*) (5)

EpiOcular™ Eye Irritation Test (OCL-200-EIT), a reconstructed human cornea-like epithelium (RhCE) test method, was conducted according to OECD guideline 492 [37]. The EpiOcular™ human cell construct used in the assay was purchased from MatTek Corporation (Bratislava, Slovakia). First, test materials were applied topically to the model, and the irritation potentials of the test materials were evaluated by the relative viability of the treated tissues relative to the negative control-treated tissues, which were determined by the NAD(P)H-dependent microsomal enzyme reduction of MTT (Equation (4)). The data are presented as relative viability. When meeting all the acceptance criteria, the final tissue viability above 60% relative to negative control-treated tissue viability is labeled as non-irritant, and below 60% is labeled as an irritant.

### 2.6. Statistical Analysis

The data are depicted as mean ± SEM. Statistical significance of differences was determined by a one-sample t test or one-way analysis of variance (ANOVA) followed by a Bonferroni post-hoc analysis and was considered significant at *p* ≤ 0.05. Statistical analysis was performed using GraphPad Prism 5.0 (GraphPad software, La Jolla, CA, USA).

## 3. Results and Discussion

### 3.1. Formation of Particles with Different Sizes

Nanoprecipitation and single emulsion approaches were applied to obtain PLA- and PCL-based nanoparticles (NPs) and microparticles (MPs), correspondingly [19,31]. The particles obtained showed different hydrodynamic sizes, ζ-potentials, encapsulation efficiencies, and particles distributions (Table 1).

The single emulsion method [14,38] is based on dispersing the polymer solution in hydrophobic solvent in excess of the water phase containing surfactants to prevent the fast coalescence of oil drops. The evaporation of organic solvent leads to the formation of polymer particle suspension. The model drug—prednisolone—was added to the polymer solution before the emulsification. To minimize polymer phase separation upon its contact with water and to obtain the fine particles, the SDS/PVA stabilizing mixture was applied for particle formation [14].

To formulate the particles of a smaller size, the procedure of the nanoprecipitation method was used. In this case, THF, a water-miscible solvent, was employed as an organic phase for the ability to solubilize both the polymers and the drugs. Good water miscibility of THF allows fast dispersion of the polymer due to rapid diffusion of THF into the water. Applying appropriate water/THF ratios [31] allows the formation of fine polymer dispersion in water. Overall, the preparation methods reported here appeared to be suitable for the formulation of prednisolone-loaded MPs and NPs based on both PLA and PCL.

**Figure 2:**
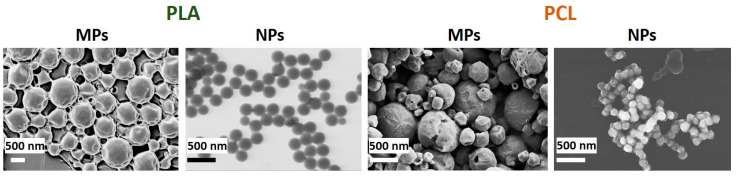
SEM and STEM (for PLA NPs) images of particles prepared via emulsion evaporation and nanoprecipitation techniques. PLA = poly(L-lactic acid), PCL = poly(ε-caprolactone), MPs = microparticles, NPs = and nanoparticles.

Particles obtained by the emulsion approach possess a greater size than those prepared via nanoprecipitation and labeled as microparticles (MPs) and nanoparticles (NPs), respectively (Table 1, Figure 2). Remarkably, the data on the particles’ size and size distribution showed a clear dependence on polymer hydrophobicity that was in complete agreement with our previous results [14]. ζ-potential of both PLA and PCL MPs, and NPs is negative, which is due to the presence of carboxylic groups on their surface. The contact angles of corresponding polyester-based films (Table 1) indicate a greater hydrophobicity of PCL-based materials as compared to PLA-based materials. However, both types of material possess contact angle less than 90°, so they could be classified as hydrophilic ones.

The relevant differences in entrapment efficiency were observed for microparticle formulations. The highest entrapment efficiency (EE) was observed for more hydrophobic PCL, while the EE was relatively low in the case of PLA with lower hydrophobicity (Table 1). It was observed that the increase in polymer hydrophobicity led to a more significant entrapment efficiency of a model drug. Similar observations were reported earlier [29]. In the published study, the effect of polymer hydrophobicity on a model hydrophobic drug, namely, risperidone loading and release from microparticles formulated on the base of PLA and PCL polymers, was presented. Our results come in accordance with this study. Overall, the EE of NPs exceed those of MPs. This might be caused by a sharper polymer phase separation in nanoprecipitation compared to the emulsion-based approach. The latter approach can be characterized by the presence of oil drops in water medium, so some drug can leak out from the particle-forming organic phase to the dispersing aqueous phase. In the case of nanoprecipitation, rapid phase separation leads to immediate particle suspension formation, leading to the entrapment of a higher amount of the drug.

### 3.2. Formation of Particles with a Tunable Surface Charge

It is well known that increasing particle charges decrease their aggregation due to electrostatic repulsion [39]. Moreover, the particle charge is also crucial for their biological properties [19,38]. In the current study, two biodegradable polyelectrolytes, namely, PLys and Hep, were applied to alter the surface charge of the particles (Figure 2). In our previous study, the covalent/electrostatic attachment of PLys/Hep, respectively, onto the particles surface, was performed to hydrophilize the surface and to study their impact on particles’ biodistributions and intracellular uptake into different cell types [19].

In the current study, we were aimed to prepare the particles with different hydrophilicity and charged layers on the surface to investigate their effect on drug release. The attachment of PLys changes all particles’ surface charge to remarkably positive values as compared to the initial particles (Figure 1, Table 1). Additional electrostatic attachment of Hep leads to the formation of a negatively charged surface (Figure S1 in Supplementary Materials). This proves the successful attachment of the charged molecules to particle surfaces. The high absolute values of ζ-potential result in increased cationic and anionic charge density on the surface of modified particles. Moreover, it was shown that interpolyelectrolyte layer thickness could be controlled by sequential covering of negatively charged particles with polycation and then by polyanion again. It is notable that charge inversion was performed by rapid addition of macromolecules with the opposite charge. Thus, the aggregation of particles at a zero charge point was escaped.

The formation of polyelectrolyte layers on the particle surface has led to the increase of the particle hydrodynamic diameter (Table 1). At the same time, the particle distribution was not growing drastically and the particles remain quite stable. All non-modified and modified polyester-based particles showed a unimodal and quite a narrow size distribution, revealing their stability and uniformity (Figures S2 and S3 in Supplementary Materials). Only PCL NPs modified by PLys and Hep showed some aggregation, possibly due to corona-forming macromolecules interweaving during purification and lyophilization. It should be noted here that all types of particles possess a good ability to redisperse after lyophilization.

The contact angle of model polyester-based films decrease upon modification with PLys-Hep interpolyelectrolyte layers, indicating more hydrophilic properties of the surface. Thus, it could be assumed that attachment of polyelectrolyte corona increases the positive interaction with water.

The EE of prednisolone for MPs and NPs modified with PLys and Hep were lower than that for initial particles (Table 1). This fact could be explained by the effect of a particle modification process. Such a process involves partial hydrolysis of particle surface in the presence of sodium hydroxide and covalent attachment of PLys. These stages directly affect the surface layer of the particles and involve many centrifugation steps, so some drug leakage from the particles’ surface seems to be very probable.

### 3.3. Release Kinetics of Prednisolone

**Figure 3:**
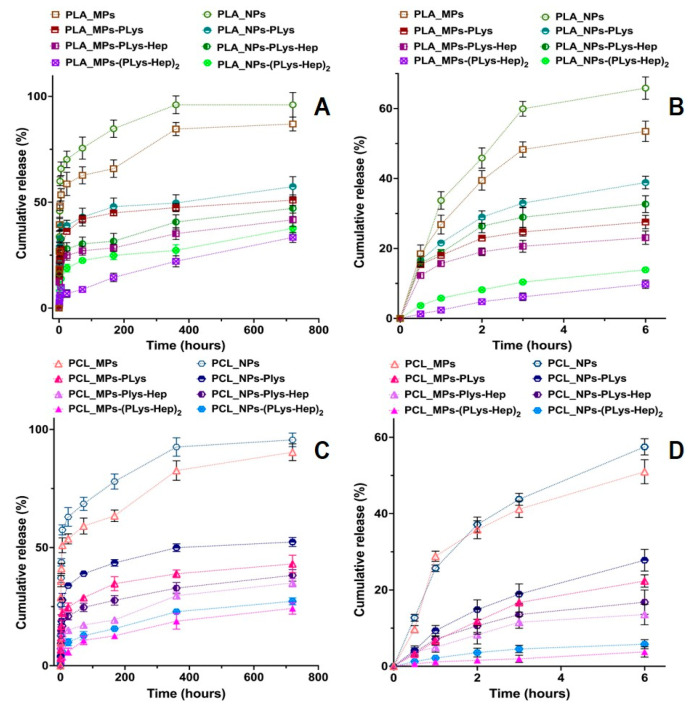
Kinetic curves of prednisolone release from the non-modified and modified particles based on PLA (**A**,**B**) and PCL (**C**,**D**) particles during 720 h (**A**,**C**) and 6 h (**B**,**D**). Conditions: 0.01 M phosphate-buffered saline (PBS), pH 7.4, containing 0.1% of SDS. Fitting of prednisolone release data are presented in Supplementary Materials.

The objective of this study was to elucidate the effect of surface properties of polyesters-based micro- and nanoparticles with different hydrophobicities on the release kinetics of the encapsulated model drug prednisolone. This corticosteroid was selected as a model drug for future studies because it was extensively used in treatments of numerous inflammatory eye diseases [4,26,40], either in the form of eye drops or ointment. The in vitro prednisolone release profiles during 720 h (Figure 3A,C) and 6 h (Figure 3B,D) clearly show the effect of both MPs and NP surface modification on the drug dissolution kinetics. One can observe the more rapid release from NPs as compared to MPs. This could be explained by the greater specific surface area of NPs, which allows the more rapid drug dissolution. Moreover, fast release from NPs is favored by a lesser polyester thickness, through which the diffusion of the drug occurs, and greater drug concentration in the particles (drug loading, see Table 1).

**Figure 4:**
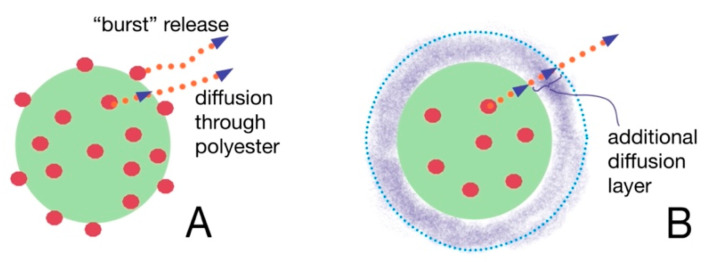
The scheme showing the difference between drug release from non-modified (**A**) and modified (**B**) particles.

The prednisolone release from modified particles visibly differs from that observed for initial particles. Moreover, the increase in polyelectrolyte layer thickness on the surface of polyester-based particles diminishes the rate of release. It is also interesting that non-modified PLA/PCL-based MPs and NPs possess an initial “burst” drug release stage. More than 50% of prednisolone rapidly dissolves at this stage during first 2–3 h (Figure 3B,C). Introduction of PLys- and Hep-based polyelectrolyte layers on the surface of the particles minimize such a “burst” release stage (Figure 3). Notably, the particles covered by two layers of polyelectrolytes, (PLys-Hep)_2_, show even a release profile, without an initial “burst”. This could be referred to the action of two factors, namely, a decrease in the drug loading due to leakage of the drug during modification and formation of the hydrophilic layer on the particle surface. This layer could serve as an additional barrier for drug diffusion, which helps to prolong the drug release (Figure 4).

Interestingly, prednisolone release was faster from modified NPs as compared to modified MPs. This should be generally related to a greater specific surface area of the particles with a smaller diameter, but also could be provoked by a faster degradation of NPs promoted by surface hydrophilization and swelling of the corona layer. However, the effect of polyelectrolyte surface layers on degradation should be studied in future experiments.

Generally, the obtained prednisolone formulations based on modified PLA/PCL MPs and NPs allowed a sustained release of the encapsulated drug for more than 30 days. Previous studies by Budhian et al. [41] showed that in vitro haloperidol release from PLGA/PLA particles is affected by particle size and coating. The authors showed a slower release for particles with a greater hydrophobicity and elevated particle size. It was also demonstrated that chitosan-coated particles considerably reduce the initial “burst” stage. In a similar study, the reduction in the initial “burst” stage of lidocaine release from PLGA microspheres by coating them with chitosan was reported [42]. In the mentioned studies, the coating of particles with hydrophilic corona was performed only via electrostatic interactions between the coating polymer and polyester. Neither was covalent attachment of polyelectrolyte, nor variation of polyelectrolyte layer thickness, performed in these studies. Notably, our results come in accordance with previous findings irrespective of the nature of coating layers on the surface of particles and the method of biopolymer attachment. However, in our work, the higher charge density, covalent attachment of PLys to the particle surface and the possibility to form several polyelectrolyte layers allowed us to achieve better control over the drug release.

In order to analyze and compare the mechanisms of drug release from non-modified and modified PLA- and PCL-based MPs and NPs, the resulting drug release profiles were approximated with the application of a number of kinetic mathematical models (Table 2, for full data see Supplementary Materials Tables S1–S4).

The first observation within the results presented in Table 2 was that diffusion-controlled zero-order release kinetics is not possible in the case of non-modified particles, as well as in the case of particles with one polyelectrolyte layer. The good fitting with this model was achieved for MPs with two PLys-Hep layers. Thus, it could be concluded that such formulations can release drugs within the first six hours according to the zero-order kinetic law. However, on the long scale (see Tables S1–S4), the release becomes dependent on the concentration of drugs in the particles and the first-order release model works better. One can also observe the decrease in both K_0_ and K_1_ in the case of modified particles. The constant values decrease with the increase in polyelectrolyte layer thickness, indicating the role of surface hydrogel corona in the reduction of the drug dissolution rate.

Approximation of release data with Higuchi, Hixson-Crowell, Baker-Lonsdale and Hopfenberg models allows the conclusion that best fitting is observed in the case of Baker-Lonsdale and Higuchi models, while the Hixson-Crowell and Hopfenberg models are less applicable (Table 2). Thus, it could be concluded that the dominating mechanism of prednisolone release is diffusion, but not drug dissolution or polyester degradation. In addition, it is quite noticeable that applied models are a better fit release from non-modified particles and particles with double PLys-Hep corona, but not from intermediate particulate formulations. Thus, these particles could be considered as two extreme cases.

Application of parametric models, such as Korsmeyer–Peppas, Peppas–Sahlin and Weibull, allows the best fitting of the obtained experimental data (Table 2). The determined coefficients are very close to 1.0. Thus, the calculated parameters could be considered as confident. The values of the n parameter from the Korsmeyer–Peppas model confirms that the main mechanism of drug release is Fickian diffusion. Only in the case of PLA MPs-(PLys-Hep)_2_ is the n value above 0.7, revealing the anomalous diffusion, when diffusion and polymer relaxation are both important factors. The Peppas–Sahlin model shows the ratio of diffusion (K_1_) and relaxation of polymer molecules (K_2_) as factors of drug release. The diffusion is always more important for non-modified particulate formulations (K_1_ > K_2_). However, the modification of particle surface increases the role of relaxation for PCL and PLA MPs-(PLys-Hep)_2_ K_1_ = K_2_. The possible explanation for this result is that the relaxation of polyelectrolytes within surface hydrogel corona becomes an important factor affecting the release of prednisolone.

The Wibull model contains two parameters, α (time scale factor) and β (release curve factor). The first factor increases upon modification of the surface, revealing a more prolonged release. The second parameter is always below 1.0, which is characteristic for parabolic release curve [43].

Thus, the performed study of prednisolone release from polyester particles modified by PLys and Hep interpolyelectrolyte hydrogel corona allows the conclusion that the release is affected by particle size and surface modification. The smaller the size of the particles, the greater the release rate that could be expected. As the main mechanism of drug release during the first month is diffusion, the surface hydrogel layer will definitely affect the release rate, making it more sustained.

Here we should note that in current studies we have performed the release studies in enzyme-free medium to compare the release kinetics in similar conditions. The presence of specific enzymes within the tissues will affect the fate of such particles and drug release kinetics.

### 3.4. Effect of Particles Surface Modification on Their Cytotoxicity

**Figure 5:**
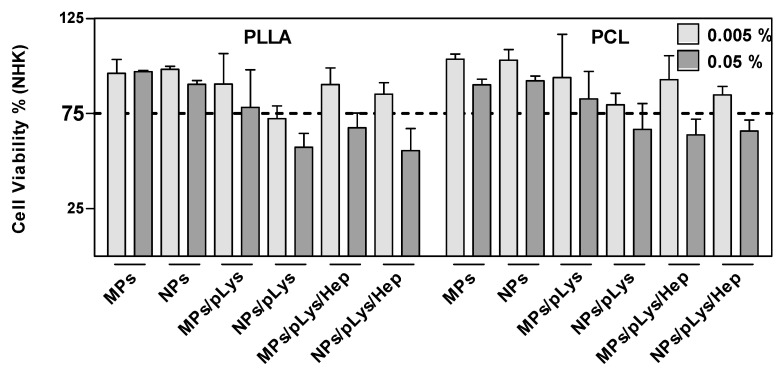
Effect of polyester particles on cell viability. NHK were exposed to the indicated concentrations for 24 h. A declined formazan formation <75% after MTT exposure indicates cytotoxicity. Data are presented as mean ± SD, *n* = 3.

All particles were incubated with human NHK cells at the concentrations of 0.005 and 0.05% for 24 h and showed a concentration-dependent cytotoxic effect where a higher concentration led to more cytotoxic effects (Figure 5). Most of the modified particles, regardless of PLys or Hep, showed no cytotoxic effects at lower concentrations, which indicates the good biocompatibility of the particles, although PLA/polylysine nanoparticles exhibiting surface charges showed a cytotoxic effect. This may be caused by the fact that the cell surface is negatively charged, and the polylysine-modified cationic nanoparticles readily penetrated through the cell membrane [44,45], which may lead to the cytotoxic effect. All modified particles presented more cytotoxic effects than PLA or PCL particles alone, which may be due to the fact that the amphiphilic modification conjugates could better diffuse through the bilayered lipid membranes [46]. Except for the PLA-MPs and PCL-NPs, where similar viabilities were detected, all other microparticles presented better biocompatibility than nanoparticles. This could be due to the smaller size, which could enhance the internalization by the endocytic mechanism and induce cytotoxicity upon uptake [47].

Furthermore, as the stimulation period for cytotoxicity study is 24 h and for irritation studies only 10 or 30 min, the cumulative releases of the model drug was negligible (Figure 3).

### 3.5. Modified Particles Irritation Potential

**Figure 6:**
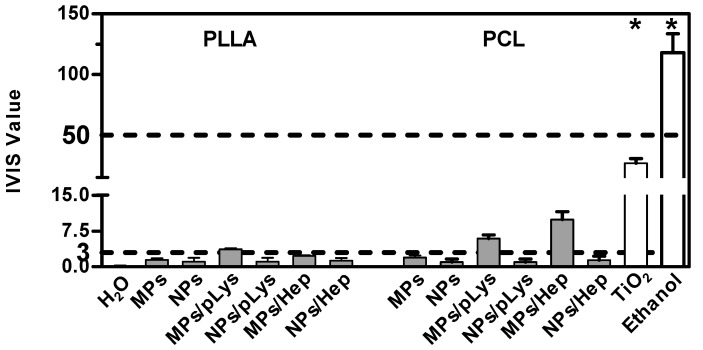
Eye irritation potential of polyester particles at the concentration of 0.5% by BCOP assay. Mean ± SD, *n* = 3. * *p* ≤ 0.05, ANOVA with post hoc Bonferroni test.

The RBC test, as a general irritancy potential test, was performed and evaluated on the basis of the hemoglobin leakage followed by the disruption of the cell membranes. Under the standardized exposure time of 10 min, all samples showed negligible hemolysis, with the highest hemolysis value at around 4% by PLA MPs (Figure 6), far below the irritancy effect rate, which indicates the good tolerability of the polyester particles on a cell monolayer level.

The RBC test, which is rapid and inexpensive, is normally used for predicting the eye irritancy of surfactants [48]. However, toxicity evaluations should be confirmed on a tissue analysis level, preferably on the skin or cornea tissues instead of only on isolated cells, as the interested drug delivery system would be used for topical applications. The BCOP assay and EpiOcular™ test, both validated alternative test systems, were combined in the aim of replacing the original reference method, in vivo Draize eye irritation test, to predict the eye irritation potential.

According to the OECD guidelines 437 and 492, both the BCOP assay and the EpiOcular™ test were the required methods of the chemical legislation. However, the publications or legislations regarding the nanomaterials were almost absent. The Draize test is traditionally applied on live rabbits, and the accuracy is relatively low, despite the raised animal ethical issues. Thus, a more accurate and alternative evaluation system seems to be of prompt demands. The BCOP assay could distinguish the identification of substances from “No Category” and “Category 1”, and an EpiOcular test could be distinguished from “No Category” and “Category 1 or 2”. A combination of these two tests could provide an evaluation strategy that covers the full spectrum of eye irritation potentials. The two-tiered system has been established and tested for 23 metallic nanoparticles and carbon nanotubes, so we have adopted this strategy and extended its use onto polyester particles.

**Figure 7:**
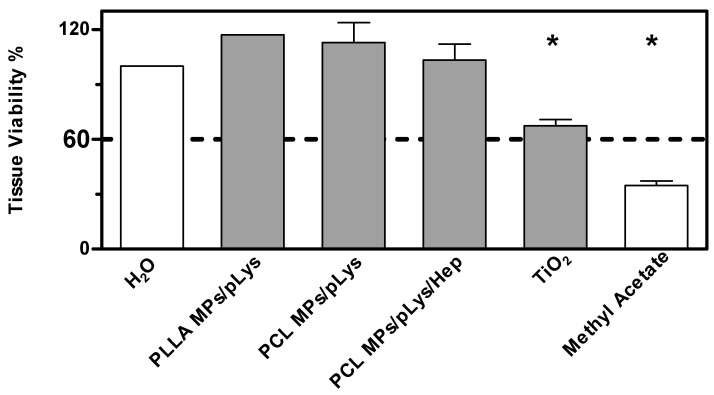
Eye irritation potential of polyester particles at the concentration of 0.5% by the EpiOcular test. Mean ± SD, *n* = 3. * *p* ≤ 0.05, ANOVA with post hoc Bonferroni test.

As a more toxic effect was shown at the higher concentration in the previous tests on cells, both assays were performed with all samples at the concentration of 0.5%. The BCOP assay was first performed due to its simplicity and inexpensiveness, and an in vitro irritation score (IVIS) was calculated to classify the test substance into “No Category” (≤3), “No prediction can be made” (>3; ≤55) and “Category 1”, inducing serious eye damage (>55), as defined by the United Nations Globally Harmonized System of Classification and Labelling of Chemicals (UN GHS). As shown in Figure 7, distilled water and methyl acetate were applied as negative and positive controls, and titanium dioxide nanomaterials as a nanoparticulate reference. PLA MPs-PLys, PCL MPs-PLys, PCL MPs-PLys-Hep and TiO_2_ were categorized as “no prediction can be made”, while all other particles fell into the “no category”, suggesting no eye irritation potential. Subsequently, the EpiOcular test was performed to distinguish substances when no prediction can be made. The final evaluating criteria for the EpiOcular test is tissue viability, where above 60.0% is relative to the negative control non-irritant and below 60.0% is an irritant. All the particles presented a tissue viability above 60%, indicating that no irritancy can be predicted, although TiO_2_ nanoparticles showed a broader line irritancy [30]. Combining the results from both assays, all polyester particles were predicted to not be an eye-irritant, which is in accordance with the RBC test result.

## 4. Conclusions

Polyester-based micro- and nanoparticles were successfully prepared and characterized as carriers for the prednisolone model drug. The formulated particles were modified with biodegradable polyelectrolytes to tune the surface charge. The formed polyelectrolyte-based hydrogel corona affected the release kinetics of the prednisolone drug. The release of the drug from modified polyester particles was shown to proceed for more than 30 days. The possibility to control the release rate by adjusting the coating layer was shown. Based on the comparison of model approximations, the prednisolone release process was mainly governed by molecular diffusion as the principal mechanism. The developed particle formulations were found to be nontoxic in the tested range of concentrations. A two-tier strategy with the EpiOcular^TM^ Eye Irritation Test, as well as Bovine Corneal Opacity and the Permeability Assay, was adopted for an eye irritancy evaluation. We found no eye-irritant potential for any polyester carriers in this study. Hence, the family of polyester micro- and nanocarriers, with varied size, surface charge and hydrophilicity, is a good indicator to gain insights into the influential factors on particulate functionality and risk, and loaded with a prednisolone model drug (something understandable) it is promising for further investigation as a biocompatible ocular drug delivery carrier.

## Figures and Tables

**Table 1:** Characteristics of PLA- and PCL-based particles. PLA = poly(L-lactic acid), PCL = poly(ε-caprolactone), MPs = microparticles, NPs = and nanoparticles.

Polymer Type	Particles Type	d_H_^DLS^, (nm)	PDI	ζ-potential, (mV)	Entrapment Efficiency, (%)	DL, (μg/mg of Particles)	Films Contact Angle, (°)
PLA	MPs	678 ± 46	0.10	−40 ± 2	9	14	68.6 ± 0.8
	NPs	178 ± 10	0.06	−42 ± 1	93	86	
	MPs/PLys	720 ± 100	0.14	46 ± 4	6	9	55.9 ± 0.6
	NPs/PLys	210 ± 90	0.43	32 ± 7	76	91.45	
	MPs/PLys/Hep	820 ± 130	0.16	−35 ± 4	8	7	45.3 ± 0.5
	NPs/PLys/Hep	290 ± 40	0.14	−15 ± 8	59	41	
	MPs/(PLys/Hep)_2_	908 ± 170	0.19	−28 ± 4	8	7	38.3 ± 2.2
	NPs/(PLys/Hep)_2_	303 ± 75	0.25	−12 ± 2	59	40	
PCL	MPs	990 ± 98	0.11	−22 ± 1	16	17	83.9 ± 0.9
	NPs	188 ± 12		−27 ± 1	95	102	
	MPs/PLys	1070 ± 20	0.02	39 ± 4	17	21	58.1 ± 0.7
	NPs/PLys	230 ± 80	0.35	30 ± 2	83	114	
	MPs/PLys/Hep	1084 ± 70	0.07	−30 ± 5	10	15	47.8 ± 0.5
	NPs/PLys/Hep	280 ± 40	0.14	−22 ± 3	42	42	
	MPs/(PLys/Hep)_2_	1192 ± 114	0.10	−21 ± 5	9	13	41.2 ± 1.2
	NPs/(PLys/Hep)_2_	337 ± 108	0.32	−18 ± 3	40	39	

**Table 2:** Correlation coefficients and constants evaluated by fitting prednisolone release profiles during first six hours of release. The models used for drug release fitting, full curves fitting data and linearization curves are presented in Supplementary Materials (Tables S1–S4). PLA = poly(L-lactic acid), PCL = poly(ε-caprolactone), MPs = microparticles, NPs = and nanoparticles.

Model	Para-meter	MPs	MPs-PLys	MPs-PLys-Hep	MPs-(PLys-Hep)_2_	NPs	NPs-PLys	NPs-PLys-Hep	NPs-(PLys-Hep)_2_
PLA									
Zero-order	**R^2^**	0.8707	0.7769	0.7786	**0.9839**	0.8801	0.8552	0.8222	0.9414
	**K_0_**	11.6	6.2	5.2	1.8	14.1	8.4	7.2	2.8
First-order	**R^2^**	0.9396	0.8114	0.8063	**0.9867**	0.9662	0.8981	0.8618	0.9494
	**K_1_**	0.19	0.08	0.06	0.02	0.28	0.12	0.10	0.03
Higuchi	**R^2^**	0.9774	0.9360	0.9375	**0.9816**	0.9738	0.9758	0.9598	**0.9985**
Hixson-Crowell	**R^2^**	0.9468	0.7999	0.7970	**0.9858**	0.9468	0.8843	0.8487	0.9468
Baker-Lonsdale	**R^2^**	**0.9866**	0.9455	0.9450	**0.9815**	**0.9819**	**0.9839**	0.9687	**0.9987**
Hopfenberg	**R^2^**	0.9396	0.8114	0.8063	**0.9867**	0.9659	0.8980	0.8617	0.9493
Korsmeyer-Peppas	**R^2^**	**0.9869**	**0.9973**	**0.9892**	**0.9835**	**0.9775**	**0.9973**	**0.9940**	**0.9985**
	**n**	0.40	0.23	0.24	0.73	0.44	0.33	0.29	0.50
Peppas-Sahlin	**R^2^**	**0.9996**	**0.9993**	**0.9999**	**0.9994**	**0.9970**	**0.9998**	**0.9981**	**0.9998**
	**K_1_**	32.2	23.7	19.8	6.1	35.2	26.4	24.7	6.3
	**K_2_**	4.8	5.0	4.2	8.7	4.6	4.3	4.6	0.6
Weibull	**R^2^**	**0.9950**	**0.9984**	**0.9997**	**0.9992**	**0.9956**	**0.9991**	**0.9961**	**0.9997**
	**α**	2.5	4.5	5.3	29.9	1.9	3.6	4.0	5.3
	**β**	0.4	0.2	0.2	0.6	0.4	0.3	0.3	0.2
**PCL**									
Zero-order	**R^2^**	0.8707	0.7769	0.7786	**0.9839**	0.8801	0.8552	0.8222	0.9414
	**K_0_**	11.6	6.2	5.2	1.8	14.1	8.4	7.2	2.8
First-order	**R^2^**	0.9336	0.9731	0.9346	**0.9897**	0.9726	**0.9801**	0.9378	0.9409
	**K_1_**	0.17	0.05	0.03	7 × 10^−3^	0.19	0.06	0.04	0.01
Higuchi	**R^2^**	0.9690	0.9844	0.9867	0.9731	**0.9918**	**0.9904**	**0.9898**	**0.9913**
Hixson-Crowell	**R^2^**	0.9176	0.9696	0.9315	**0.9896**	0.9600	0.9765	0.9339	0.9397
Baker-Lonsdale	**R^2^**	0.9742	**0.9830**	**0.9871**	0.9751	**0.9927**	**0.9884**	**0.9904**	**0.9918**
Hopfenberg	**R^2^**	0.9335	0.9731	0.9346	**0.9897**	0.9726	**0.9801**	0.9378	**0.9897**
Korsmeyer-Peppas	**R^2^**	0.9716	**0.9907**	**0.9866**	**0.9973**	**0.9919**	**0.9961**	**0.9898**	**0.9914**
	**n**	0.44	0.65	0.51	0.42	0.49	0.64	0.51	0.53
Peppas-Sahlin	**R^2^**	**0.9844**	**0.9979**	**0.9960**	**0.9967**	**0.9976**	**0.9998**	**0.9992**	**0.9999**
	**K_1_**	27.1	53.5	6.0	0.8	26.8	33.2	7.6	2.4
	**K_2_**	3.6	60.6	0.7	0.9	3.1	48.8	0.9	0.3
Weibull	**R^2^**	**0.9991**	**0.9972**	**0.9896**	**0.9938**	**0.9999**	**0.9999**	**0.9985**	**0.9976**
	**α**	2.5	10.1	14.4	105.8	2.7	8.3	10.5	33.8
	**β**	0.3	0.6	0.5	0.8	0.5	0.6	0.4	0.4

## Data Availability

The prednisolone data approximations are available in excel format at google drive: https://drive.google.com/drive/folders/1Rre1REwvMYzEUpFPx2DOC9CE3JS0Tp2N?usp=sharing (accessed on 15 April 2021).

## Supplementary Materials

The following are available online at https://www.mdpi.com/article/10.3390/pharmaceutics13060801/s1, Figure S1. ζ-potential diagrams obtained by ELS (Zetasizer Nano ZS, Malvern) for non-modified and modified PLA NPs; Figure S2. Size distribution diagrams obtained by DLS (Zetasizer Nano ZS, Malvern) for non-modified and modified PLA MPs and NPs; Figure S3. Size distribution diagrams obtained by DLS (Zetasizer Nano ZS, Malvern) for non-modified and modified PCL MPs and NPs; Table S1. Correlation coefficients and constants evaluated by fitting prednisolone release from PLA MPS; Table S2. Correlation coefficients and constants evaluated by fitting prednisolone release from PLA NPS; Table S3. Correlation coefficients and constants evaluated by fitting prednisolone release from PCL MPS; Table S4. Correlation coefficients and constants evaluated by fitting prednisolone release from PCL NPS.
