# Supplementary material for: Effect of Poly(L-lysine) and Heparin Coatings on the Surface of Polyester-Based Particles on Prednisolone Release and Biocompatibility
Source: Pharmaceutics. 2021 May 27;13(6):801. doi: 10.3390/pharmaceutics13060801 (PMC8229182; doi:10.3390/pharmaceutics13060801)
Supplement: Supplementary file 1 [file pharmaceutics-13-00801-s001.zip › pharmaceutics-1220663-SI.pdf]

pharmaceutics

MDPI

Supplementary Materials: Effect of Poly(L-lysine) and Heparin Coatings on the Surface of Polyester-Based Particles on Prednisolone Release and Biocompatibility

Abdelrahman Mohamed [1,2], Viktor Korzhikov-Vlakh [1,*], Nan Zhang [3], André Said [3], Julia Pilipenko [1], Monika Schäfer-Korting [3], Christian Zoschke [3,4] and Tatiana Tennikova [1]

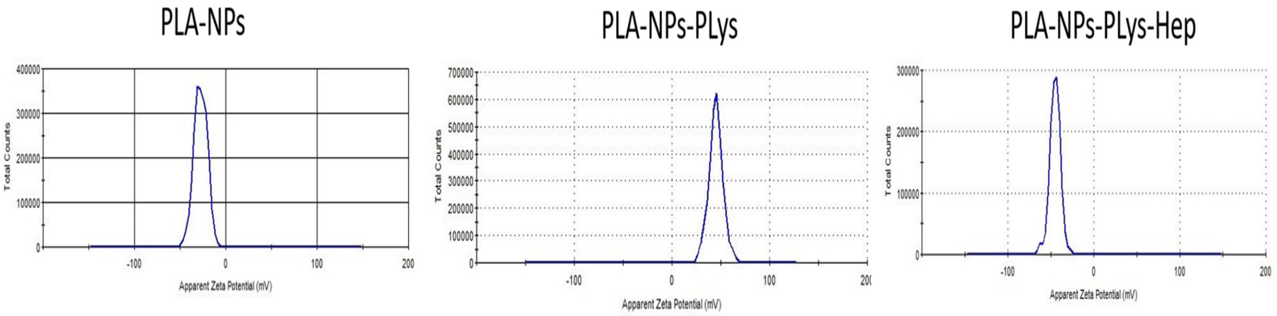

Figure S1. ζ-potential diagrams obtained by ELS (Zetasizer Nano ZS, Malvern) for non-modified and modified PLA NPs.

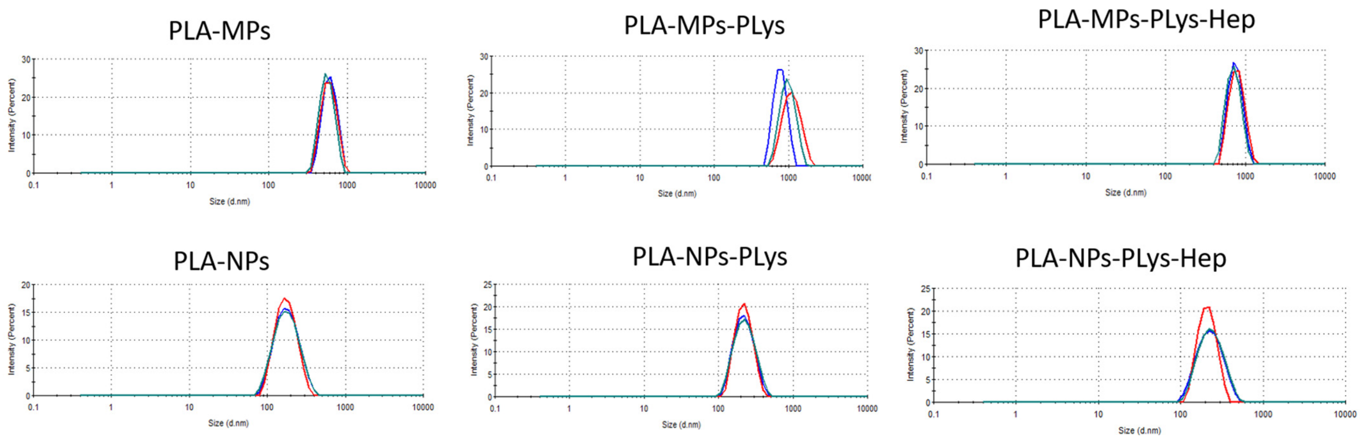

Figure S2. Size distribution diagrams obtained by DLS (Zetasizer Nano ZS, Malvern) for non-modified and modified PLA MPs and NPs.

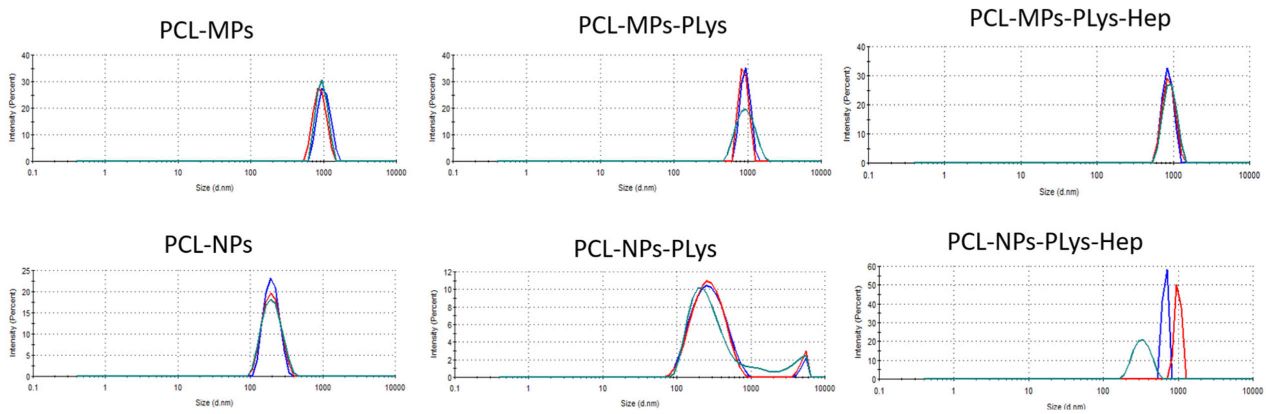

Figure S3. Size distribution diagrams obtained by DLS (Zetasizer Nano ZS, Malvern) for non-modified and modified PCL MPs and NPs.

Table S1. Correlation coefficients and constants evaluated by fitting prednisolone release from PLA MPS.

Model	PLA MPs		PLA MPs-PLys		PLA MPs-PLys-Hep		PLA MPs-(PLys-Hep)2	
	6 h	***720 h***	***6 h***	***720 h***	*6 h*	***720 h***	*6 h*	***720 h***
Zero-order<br>$F = K_0*t$	R^2 = 0.8707<br>K_0 = 11.561	R^2 = 0.7123<br>K_0 = 0.162	R^2 = 0.7769<br>K_0 = 6.194	R^2 = 0.6929<br>K_0 = 0.096	R^2 = 0.7786<br>K_0 = 5.182	R^2 = 0.7723<br>K_0 = 0.074	R^2 = **0.9839**<br>K_0 = 1.792	R^2 = 0.9581<br>K_0 = 0.052
First-order<br>$F = 100*[1-Exp(-K_1*t)]$	R^2 = 0.9396<br>K_1 = 0.194	R^2 = 0.9256<br>K_1 = 0.178	R^2 = 0.8114<br>K_1 = 0.079	R^2 = 0.7603<br>K_1 = 0.002	R^2 = 0.8063<br>K_1 = 0.063	R^2 = 0.7964<br>K_1 = 0.001	R^2 = **0.9867**<br>K_1 = 0.019	R^2 = 0.9647<br>K_1 = 0.001
Higuchi<br>$F = K_H*t^{0.5}$	R^2 = 0.9774<br>K_H = 24.837	R^2 = 0.8349<br>K_H = 4.366	R^2 = 0.9360<br>K_H = 13.741	R^2 = 0.8385<br>K_H = 2.623	R^2 = 0.9375<br>K_H = 11.492	R^2 = 0.8638<br>K_H = 1.954	R^2 = **0.9816**<br>K_H = 3.588	R^2 = 0.9729<br>K_H = 1.221
Korsmeyer-Peppas<br>$F = K_{KP}*t^n$	R^2 = **0.9869**<br>K_{KP} = 28.327<br>n = 0.389	R^2 = 0.9690<br>K_{KP} = 34.072<br>n = 0.147	R^2 = **0.9973**<br>K_{KP} = 18.620<br>n = 0.234	R^2 = **0.9892**<br>K_{KP} = 20.814<br>n = 0.144	R^2 = **0.9971**<br>K_{KP} = 15.504<br>n = 0.238	R^2 = **0.9835**<br>K_{KP} = 16.152<br>n = 0.134	R^2 = **0.9973**<br>K_{KP} = 2.678<br>n = 0.733	R^2 = 0.9678<br>K_{KP} = 2.141<br>n = 0.405
Hixon-Crowell<br>$F=100*[1-(1-K_{HC}*t)^3]$	R^2 = 0.9468<br>K_{HC} = 0.077	R^2 = 0.8161<br>K_{HC} = 1.9*10^{-3}	R^2 = 0.7999<br>K_{HC} = 0.024	R^2 = 0.7351<br>K_{HC} = 4.9*10^{-4}	R^2 = 0.7970<br>K_{HC} = 0.020	R^2 = 0.7886<br>K_{HC} = 3.3*10^{-4}	R^2 = **0.9858**<br>K_{HC} = 0.006	R^2 = 0.9629<br>K_{HC} = 2.0*10^{-4}
Hopfenberg<br>$F=100*[1-(1-K_{HB}*t)^n]$	R^2 = 0.9396<br>K_{Hfb} = 9.1*10^{-5}	R^2 = 0.9258<br>K_{Hb} = 6.8*10^{-5}	R^2 = 0.8114<br>K_{Hb} = 6.2*10^{-5}	R^2 = 0.7603<br>K_{Hb} = 7.3*10^{-7}	R^2 = 0.8063<br>K_{Hfb} = 6.3*10^{-5}	R^2 = 0.7964<br>K_{Hfb} = 1.4*10^{-6}	R^2 = **0.9867**<br>K_{Hfb} = 1.1*10^{-4}	R^2 = 0.9647<br>K_{Hfb} = 1.4*10^{-6}
Baker-Lonsdale<br>$3/2*[1-(1-F/100)^{(2/3)}]-F/100=K_{BL}*t$	R^2 = **0.9866**<br>K_{BL} = 0.014	R^2 = 0.8795<br>K_{BL} = 6.9*10^{-4}	R^2 = 0.9455<br>K_{BL} = 3.6*10^{-3}	R^2 = 0.8636<br>K_{BL} = 1.8*10^{-4}	R^2 = 0.9450<br>K_{BL} = 2.5*10^{-3}	R^2 = 0.8712<br>K_{BL} = 8.5*10^{-5}	R^2 = **0.9815**<br>K_{BL} = 2.2*10^{-4}	R^2 = 0.9707<br>K_{BL} = 2.8*10^{-5}
Weibull<br>$F=100*\{1-Exp[-((t-Ti)^{\beta})/\alpha]\}$	R^2 = **0.9950**<br>α = 2.462<br>β = 0.396	R^2 = **0.9844**<br>α = 2.173<br>β = 0.209	R^2 = **0.9984**<br>α = 4.483<br>β = 0.220	R^2 = **0.9963**<br>α = 4.061<br>β = 0.169	R^2 = **0.9997**<br>α = 5.278<br>β = 0.193	R^2 = **0.9847**<br>α = 5.312<br>β = 0.142	R^2 = **0.9992**<br>α = 29.940<br>β = 0.649	R^2 = 0.9701<br>α = 210.796<br>β = 0.671
Peppas-Sahlin<br>$F=K_1*t^m+K_2*t^{(2*m)}$	R^2 = **0.9996**<br>K_1 = 32.2<br>K_2 = 4.8	R^2 = 0.9723<br>K_1 = 36.4<br>K_2 = 3.7	R^2 = **0.9993**<br>K_1 = 23.7<br>K_2 = 5.0	R^2 = **0.9980**<br>K_1 = 21.6<br>K_2 = 2.3	R^2 = **0.9999**<br>K_1 = 19.8<br>K_2 = 4.2	R^2 = **0.9838**<br>K_1 = 13.2<br>K_2 = 3.1	R^2 = **0.9994**<br>K_1 = 6.1<br>K_2 = 8.7	R^2 = 0.9731<br>K_1 = 2.1<br>K_2 = 0.3

Table S2. Correlation coefficients and constants evaluated by fitting prednisolone release from PLA NPS.

Model	PLA NPs		PLA NPs-PLys		PLA NPs-PLys-Hep		PLA NPs-(PLys-Hep)2	
	6 h	***720 h***	***6 h***	*720 h*	*6 h*	*720 h*	*6 h*	*720 h*
Zero-order<br>$F = K_0*t$	R^2 = 0.8801<br>K_0 = 14.1	R^2 = 0.6468<br>K_0 = 0.18	R^2 = 0.8552<br>K_0 = 8.36	R^2 = 0.6729<br>K_0 = 0.11	R^2 = 0.8222<br>K_0 = 7.21	R^2 = 0.7028<br>K_0 = 0.08	R^2 = 0.9414<br>K_0 = 2.76	R^2 = 0.8389<br>K_0 = 0.06
First-order<br>$F = 100*[1-Exp(-K_1*t)]$	R^2 = 0.9662<br>K_1 = 0.28	R^2 = 0.9597<br>K_1 = 0.27	R^2 = 0.8981<br>K_1 = 0.12	R^2 = 0.7325<br>K_1 = 2.0*10^{-3}	R^2 = 0.8618<br>K_1 = 0.10	R^2 = 0.7247<br>K_1 = 1.0*10^{-3}	R^2 = 0.9494<br>K_1 = 0.03	R^2 = 0.8599<br>K_1 = 1.0*10^{-3}
Higuchi<br>$F = K_H*t^{0.5}$	R^2 = 0.9738<br>K_H = 30.023	R^2 = 0.7891<br>K_H = 5.055	R^2 = 0.9758<br>K_H = 18.129	R^2 = 0.7970<br>K_H = 2.864	R^2 = 0.9598<br>K_H = 15.797	R^2 = 0.7803<br>K_H = 2.240	R^2 = **0.9985**<br>K_H = 5.766	R^2 = 0.9352<br>K_H = 1.628
Korsmeyer-Peppas<br>$F = K_{KP}*t^n$	R^2 = 0.9775<br>K_{KP} = 32.403<br>n = 0.436	R^2 = 0.9539<br>K_{KP} = 41.424<br>n = 0.139	R^2 = **0.9973**<br>K_{KP} = 22.125<br>n = 0.329	R^2 = 0.9749<br>K_{KP} = 25.645<br>n = 0.122	R^2 = **0.9940**<br>K_{KP} = 20.089<br>n = 0.292	R^2 = 0.9466<br>K_{KP} = 21.692<br>n = 0.105	R^2 = **0.9985**<br>K_{KP} = 5.759<br>n = 0.501	R^2 = **0.9846**<br>K_{KP} = 7.666<br>n = 0.236
Hixon-Crowell<br>$F=100*[1-(1-K_{HC}*t)^3]$	R^2 = 0.9468<br>K_{HC} = 0.08	R^2 = 0.7793<br>K_{HC} = 2.1*10^{-3}	R^2 = 0.8843<br>K_{HC} = 0.04	R^2 = 0.7119<br>K_{HC} = 6.0*10^{-4}	R^2 = 0.8487<br>K_{HC} = 0.03	R^2 = 0.7180<br>K_{HC} = 3.9*10^{-4}	R^2 = 0.9468<br>K_{HC} = 0.01	R^2 = 0.8530<br>K_{HC} = 2.6*10^{-4}
Hopfenberg<br>$F=100*[1-(1-K_{HB}*t)^n]$	R^2 = 0.9659<br>K_{Hfb} = 6.2*10^{-4}	R^2 = 0.9598<br>K_{Hb} = 1.4*10^{-4}	R^2 = 0.8980<br>K_{Hb} = 1.1*10^{-4}	R^2 = 0.7324<br>K_{Hb} = 1.1*10^{-6}	R^2 = 0.8617<br>K_{Hfb} = 8.3*10^{-5}	R^2 = 0.7246<br>K_{Hfb} = 1.9*10^{-6}	R^2 = 0.9493<br>K_{Hfb} = 3.3*10^{-4}	R^2 = 0.8599<br>K_{Hfb} = 1.6*10^{-6}
Baker-Lonsdale<br>$3/2*[1-(1-F/100)^{(2/3)}]-F/100=K_{BL}*t$	R^2 = **0.9819**<br>K_{BL} = 0.02	R^2 = 0.8488<br>K_{BL} = 6.9*10^{-4}	R^2 = **0.9839**<br>K_{BL} = 0.01	R^2 = 0.8206<br>K_{BL} = 2.3*10^{-4}	R^2 = **0.9687**<br>K_{BL} = 4.9*10^{-3}	R^2 = 0.7874<br>K_{BL} = 1.2*10^{-4}	R^2 = **0.9987**<br>K_{BL} = 5.8*10^{-4}	R^2 = 0.9414<br>K_{BL} = 5.5*10^{-5}
Weibull	R^2 = **0.9956**	R^2 = **0.9898**	R^2 = **0.9991**	R^2 = **0.9893**	R^2 = **0.9961**	R^2 = 0.9546	R^2 = **0.9997**	R^2 = **0.9895**

$F=100*\{1-Exp[-((t-Ti)^{\beta})/\alpha]\}$	α = 1.87<br>β = 0.44	α = 1.67<br>β = 0.24	α = 3.60<br>β = 0.33	α = 3.06<br>β = 0.14	α = 4.03<br>β = 0.28	α = 3.73<br>β = 0.11	α = 5.28<br>β = 0.19	α = 11.52<br>β = 0.24
Peppas-Sahlin<br>$F=K_1*t^m+K_2*t^{(2*m)}$	R^2 = 0.9970<br>K_1 = 35.2<br>K_2 = 4.6	R^2 = 0.9671<br>K_1 = 43.3<br>K_2 = 4.9	R^2 = 0.9998<br>K_1 = 26.4<br>K_2 = 4.3	R^2 = 0.9787<br>K_1 = 28.3<br>K_2 = 3.6	R^2 = 0.9981<br>K_1 = 24.7<br>K_2 = 4.6	R^2 = 0.9754<br>K_1 = 18.3<br>K_2 = 1.9	R^2 = 0.9998<br>K_1 = 6.3<br>K_2 = 0.6	R^2 = 0.9852<br>K_1 = 7.7<br>K_2 = 0.3

Table S3. Correlation coefficients and constants evaluated by fitting prednisolone release from PCL MPS.

Model	PCL MPs		PCL MPs-PLys		PCL MPs-PLys-Hep		PCL MPs-(PLys-Hep)$_2$	
	6 h	***720 h***	***6 h***	*720 h*	*6 h*	*720 h*	*6 h*	*720 h*
Zero-order<br>$F = K_0*t$	R^2 = 0.8802<br>K_0 = 10.6	R^2 = 0.7607<br>K_0 = 0.16	R^2 = 0.9622<br>K_0 = 4.30	R^2 = 0.7613<br>K_0 = 0.08	R^2 = 0.9251<br>K_0 = 2.80	R^2 = 0.8788<br>K_0 = 0.06	R^2 = **0.9894**<br>K_0 = 0.66	R^2 = 0.9286<br>K_0 = 0.04
First-order<br>$F = 100*[1-Exp(-K_1*t)]$	R^2 = 0.9336<br>K_1 = 0.17	R^2 = 0.9029<br>K_1 = 0.13	R^2 = 0.9731<br>K_1 = 0.05	R^2 = 0.8044<br>K_1 = $1.2*10^{-3}$	R^2 = 0.9346<br>K_1 = 0.03	R^2 = 0.8980<br>K_1 = $8.0*10^{-4}$	R^2 = **0.9897**<br>K_1 = $6.7*10^{-3}$	R^2 = 0.9413<br>K_1 = $4.7*10^{-4}$
Higuchi<br>$F = K_H*t^{0.5}$	R^2 = 0.9690<br>K_H = 22.6	R^2 = 0.8694<br>K_H = 4.3	R^2 = 0.9844<br>K_H = 8.8	R^2 = 0.8894<br>K_H = 2.1	R^2 = 0.9867<br>K_H = 5.8	R^2 = 0.9516<br>K_H = 1.5	R^2 = 0.9731<br>K_H = 1.3	R^2 = 0.9931<br>K_H = 1.0
Korsmeyer-Peppas<br>$F = K_{KP}*t^n$	R^2 = 0.9716<br>K_{KP} = 24.17<br>n = 0.44	R^2 = 0.9671<br>K_{KP} = 29.6<br>n = 0.17	R^2 = 0.9907<br>K_{KP} = 7.3<br>n = 0.65	R^2 = 0.9719<br>K_{KP} = 11.4<br>n = 0.21	R^2 = 0.9866<br>K_{KP} = 5.8<br>n = 0.51	R^2 = 0.9784<br>K_{KP} = 6.6<br>n = 0.25	R^2 = 0.9973<br>K_{KP} = 1.6<br>n = 0.42	R^2 = 0.9940<br>K_{KP} = 1.0<br>n = 0.75
Hixon-Crowell<br>$F=100*[1-(1-K_{HC}*t)^3]$	R^2 = 0.9176<br>K_{HC} = 0.05	R^2 = 0.8463<br>K_{HC} = $1.9*10^{-3}$	R^2 = 0.9696<br>K_{HC} = 0.02	R^2 = 0.7898<br>K_{HC} = $3.6*10^{-4}$	R^2 = 0.9315<br>K_{HC} = 0.01	R^2 = 0.8920<br>K_{HC} = $2.4*10^{-4}$	R^2 = 0.9896<br>K_{HC} = $2.2*10^{-3}$	R^2 = 0.9372<br>K_{HC} = $1.5*10^{-4}$
Hopfenberg<br>$F=100*[1-(1-K_{HB}*t)^n]$	R^2 = 0.9335<br>K_{Hfb} = $8.1*10^{-5}$	R^2 = 0.9027<br>K_{Hb} = $3.5*10^{-5}$	R^2 = 0.9731<br>K_{Hb} = $7.0*10^{-5}$	R^2 = 0.8043<br>K_{Hb} = $9.9*10^{-7}$	R^2 = 0.9346<br>K_{Hfb} = $7.6*10^{-5}$	R^2 = 0.8979<br>K_{Hfb} = $3.3*10^{-6}$	R^2 = 0.9897<br>K_{Hfb} = $1.3*10^{-4}$	R^2 = 0.9413<br>K_{Hfb} = $6.1*10^{-7}$
Baker-Lonsdale<br>$3/2*[1-(1-F/100)^{(2/3)}]-F/100=K_{BL}*t$	R^2 = 0.9742<br>K_{BL} = 0.01	R^2 = 0.9031<br>K_{BL} = $6.9*10^{-4}$	R^2 = 0.**9830**<br>K_{BL} = $1.4*10^{-3}$	R^2 = 0.9036<br>K_{BL} = $9.8*10^{-5}$	R^2 = **0.9871**<br>K_{BL} = $5.9*10^{-4}$	R^2 = 0.9547<br>K_{BL} = $4.6*10^{-5}$	R^2 = 0.9751<br>K_{BL} = $2.9*10^{-5}$	R^2 = **0.9944**<br>K_{BL} = $1.7*10^{-5}$
Weibull<br>$F=100*\{1-Exp[-((t-Ti)^{\beta})/\alpha]\}$	R^2 = **0.9991**<br>α = 2.483<br>β = 0.321	R^2 = **0.9847**<br>α = 2.457<br>β = 0.223	R^2 = **0.9972**<br>α = 10.086<br>β = 0.555	R^2 = **0.9938**<br>α = 6.435<br>β = 0.196	R^2 = **0.9896**<br>α = 14.405<br>β = 0.448	R^2 = **0.9781**<br>α = 13.602<br>β = 0.256	R^2 = **0.9938**<br>α = 105.811<br>β = 0.771	R^2 = **0.9979**<br>α = 62.802<br>β = 0.435
Peppas-Sahlin<br>$F=K_1*t^m+K_2*t^{(2*m)}$	R^2 = **0.9844**<br>K_1 = 27.063<br>K_2 = 3.613	R^2 = 0.9681<br>K_1 = 30.978<br>K_2 = 2.131	R^2 = **0.9979**<br>K_1 = 53.486<br>K_2 = 60.640	R^2 = 0.9798<br>K_1 = 10.921<br>K_2 = 0.704	R^2 = **0.9960**<br>K_1 = 6.038<br>K_2 = 0.668	R^2 = 0.9794<br>K_1 = 5.899<br>K_2 = 0.915	R^2 = **0.9957**<br>K_1 = 0.750<br>K_2 = 0.224	R^2 = **0.9976**<br>K_1 = 1.430<br>K_2 = 0.013

Table S4. Correlation coefficients and constants evaluated by fitting prednisolone release from PCL NPS.

Model	PCL NPs		PCL NPs-PLys		PCL NPs-PLys-Hep		PCL NPs-(PLys-Hep)$_2$	
	6 h	***720 h***	***6 h***	*720 h*	*6 h*	*720 h*	*6 h*	*720 h*
Zero-order<br>$F = K_0*t$	R^2 = 0.9259<br>K_0 = 11.59	R^2 = 0.7216<br>K_0 = 0.18	R^2 = 0.9682<br>K_0 = 5.27	R^2 = 0.7319<br>K_0 = 0.10	R^2 = 0.9258<br>K_0 = 3.42	R^2 = 0.8016<br>K_0 = 0.07	R^2 = 0.9373<br>K_0 = 1.16	R^2 = 0.9040<br>K_0 = 0.05
First-order<br>$F = 100*[1-Exp(-K_1*t)]$	R^2 = 0.9726<br>K_1 = 0.19	R^2 = 0.9480<br>K_1 = 0.17	R^2 = **0.9801**<br>K_1 = 0.06	R^2 = 0.8023<br>K_1 = $2.0*10^{-3}$	R^2 = 0.9378<br>K_1 = 0.04	R^2 = 0.8323<br>K_1 = $9.8*10^{-4}$	R^2 = 0.9409<br>K_1 = 0.01	R^2 = 0.9209<br>K_1 = $5.7*10^{-4}$
Higuchi<br>$F = K_H*t^{0.5}$	R^2 = **0.9918**<br>K_H = 24.3	R^2 = 0.8550<br>K_H = 4.8	R^2 = **0.9904**<br>K_H = 10.7	R^2 = 0.8731<br>K_H = 2.6	R^2 = **0.9898**<br>K_H = 7.2	R^2 = 0.9162<br>K_H = 1.8	R^2 = **0.9913**<br>K_H = 2.4	R^2 = **0.9828**<br>K_H = 1.2
Korsmeyer-Peppas<br>$F = K_{KP}*t^n$	R^2 = 0.9919<br>K_{KP} = 24.5<br>n = 0.49	R^2 = 0.9702<br>K_{KP} = 32.8<br>n = 0.17	R^2 = **0.9961**<br>K_{KP} = 9.1<br>n = 0.64	R^2 = 0.9685<br>K_{KP} = 14.9<br>n = 0.20	R^2 = **0.9898**<br>K_{KP} = 7.1<br>n = 0.51	R^2 = **0.9855**<br>K_{KP} = 9.5<br>n = 0.21	R^2 = **0.9914**<br>K_{KP} = 2.3<br>n = 0.53	R^2 = **0.9964**<br>K_{KP} = 3.0<br>n = 0.34
Hixon-Crowell<br>$F=100*[1-(1-K_{HC}*t)^3]$	R^2 = 0.9600<br>K_{HC} = 0.05	R^2 = 0.8465<br>K_{HC} = $2.1*10^{-3}$	R^2 = 0.9765<br>K_{HC} = 0.02	R^2 = 07781<br>K_{HC} = $5.2*10^{-4}$	R^2 = 0.9339<br>K_{HC} = 0.01	R^2 = 0.8221<br>K_{HC} = $2.9*10^{-4}$	R^2 = 0.9397<br>K_{HC} = $4.0*10^{-3}$	R^2 = 0.9154<br>K_{HC} = $1.8*10^{-4}$
Hopfenberg<br>$F=100*[1-(1-K_{HB}*t)^n]$	R^2 = 0.9726<br>K_{Hfb} = $3.7*10^{-5}$	R^2 = 0.9027<br>K_{Hb} = $3.5*10^{-5}$	R^2 = **0.9801**<br>K_{Hb} = $4.6*10^{-5}$	R^2 = 0.8022<br>K_{Hb} = $2.7*10^{-7}$	R^2 = 0.9378<br>K_{Hfb} = $4.6*10^{-5}$	R^2 = 0.8322<br>K_{Hfb} = $2.6*10^{-6}$	R^2 = **0.9897**<br>K_{Hfb} = $1.3*10^{-4}$	R^2 = 0.9208<br>K_{Hfb} = $3.5*10^{-6}$
Baker-Lonsdale<br>$3/2*[1-(1-F/100)^{(2/3)}]-F/100=K_{BL}*t$	R^2 = **0.9927**<br>K_{BL} = 0.01	R^2 = 0.9061<br>K_{BL} = $6.9*10^{-4}$	R^2 = **0.9884**<br>K_{BL} = $2.1*10^{-3}$	R^2 = 0.8963<br>K_{BL} = $1.8*10^{-4}$	R^2 = 0.9904<br>K_{BL} = $9.1*10^{-4}$	R^2 = 0.9257<br>K_{BL} = $6.7*10^{-5}$	R^2 = **0.9918**<br>K_{BL} = 10^{-4}	R^2 = **0.9855**<br>K_{BL} = $2.5*10^{-5}$
Weibull<br>$F=100*\{1-Exp[-((t-Ti)^{\beta})/\alpha]\}$	R^2 = **0.9999**<br>α = 2.740<br>β = 0.488	R^2 = **0.9912**<br>α = 2.336<br>β = 0.273	R^2 = **0.9999**<br>α = 8.267<br>β = 0.569	R^2 = **0.9893**<br>α = 5.606<br>β = 0.228	R^2 = **0.9985**<br>α = 10.527<br>β = 0.394	R^2 = **0.9959**<br>α = 8.986<br>β = 0.220	R^2 = **0.9976**<br>α = 33.780<br>β = 0.418	R^2 = **0.9972**<br>α = 32.202<br>β = 0.353
Peppas-Sahlin<br>$F=K_1*t^m+K_2*t^{(2*m)}$	R^2 = **0.9976**<br>K_1 = 26.808<br>K_2 = 3.094	R^2 = 0.9781<br>K_1 = 33.189<br>K_2 = 2.905	R^2 = **0.9998**<br>K_1 = 39.805<br>K_2 = 48.796	R^2 = **0.9836**<br>K_1 = 13.738<br>K_2 = 0.915	R^2 = **0.9992**<br>K_1 = 7.561<br>K_2 = 0.851	R^2 = **0.9882**<br>K_1 = 9.425<br>K_2 = 0.543	R^2 = **0.9999**<br>K_1 = 2.430<br>K_2 = 0.254	R^2 = **0.9965**<br>K_1 = 2.865<br>K_2 = 0.033

Approximation of prednisolone release data are avalable at google drive:

https://drive.google.com/drive/folders/1Rre1REwvMYzEUpFPx2DOC9CE3JS0Tp2N?usp=sharing
